# The Effect of Alcohol Consumption on the Risk of ARDS: A Systematic Review and Meta-Analysis

**DOI:** 10.1016/j.chest.2017.11.041

**Published:** 2017-12-27

**Authors:** Evangelia Simou, Jo Leonardi-Bee, John Britton

**Affiliations:** UK Centre for Tobacco and Alcohol Studies, Division of Epidemiology and Public Health, University of Nottingham, Nottingham, UK

**Keywords:** alcohol consumption, ARDS, meta-analysis, systematic review, ALI, acute lung injury

## Abstract

**Background:**

To conduct a systematic review and meta-analysis evaluating the association between alcohol consumption and the risk of ARDS in adults.

**Methods:**

Medline, EMBASE and Web of Science were searched to identify observational studies evaluating the association between prior alcohol intake and the occurrence of ARDS among adults, published between 1985 and 2015 and with no language restriction. Reference lists were also screened. Demographic baseline data were extracted independently by two reviewers and random-effects meta-analyses were used to estimate pooled effect sizes with 95% confidence intervals. Subgroup analyses were used to explore heterogeneity.

**Results:**

Seventeen observational studies (177,674 people) met the inclusion criteria. Meta-analysis of 13 studies showed that any measure of high relative to low alcohol consumption was associated with a significantly increased risk of ARDS (OR, 1.89; 95% CI, 1.45-2.48; *I*^2^ = 48%; 13 studies); no evidence of publication bias was seen (*P* = .150). Sensitivity analyses indicated that this association was attributable primarily to an effect of a history of alcohol abuse (OR, 1.90; 95% CI, 1.40-2.60; 10 studies). Also, subgroup analyses identified that heterogeneity was explained by predisposing condition (trauma, sepsis/septic shock, pneumonia; P = .003).

**Conclusions:**

Chronic high alcohol consumption significantly increases the risk of ARDS. This finding suggests that patients admitted to hospital should be screened for chronic alcohol use.

FOR EDITORIAL COMMENT, SEE PAGE 6

ARDS is a type of acute diffuse alveolar damage with an onset within 7 days of known clinical risk factors or new/worsening respiratory symptoms. The hallmarks for ARDS are hypoxemia and bilateral opacities, using either chest radiography or CT scan.^1^ Globally, ARDS is responsible for 10.4% of all ICU admissions, and approximately 23% of patients with ARDS need mechanical ventilation.^2^ ARDS is associated with high morbidity and mortality.^3^, ^4^ A 2009 systematic review assessing the mortality of ARDS over time demonstrated an overall mortality rate of 44% and 36.2% for observational studies and random controlled trials, respectively, and found that these rates were unchanged since 1994.^5^

Risk factors for the development of ARDS and for the closely related diagnosis of acute lung injury (ALI), a term also used before definitions of ARDS were standardized in 2012,^6^ include increased age and clinical factors such as sepsis, pneumonia, aspiration, trauma, pancreatitis, shock, blood transfusions, and smoke or toxic gas inhalation.^4^, ^7^, ^8^, ^9^ Alcohol abuse has also been reported to increase the risk of ARDS,^10^, ^11^ perhaps because acute alcohol intoxication increases the risk of aspiration and pulmonary infection, while chronic alcohol ingestion disturbs both immunologic and nonimmunologic host defense mechanisms within the airway, resulting in alveolar macrophage immune dysregulation and alveolar epithelial barrier dysfunction.^12^

To date, however, there remains limited and inconsistent evidence on the relation between alcohol consumption and the risk of ARDS. To synthesize this mixed evidence to estimate an overall magnitude of risk, and to explore whether this varies by predisposing condition for ARDS, we therefore now report a systematic review and meta-analysis of observational studies of the association between alcohol consumption and ARDS.

## Methods

The PRISMA (Preferred Reporting Items for Systematic Reviews and Meta-Analyses)^13^ and MOOSE (Meta-analysis of Observational Studies in Epidemiology)^14^ guidelines were used for the conduction of this systematic review and meta-analysis (e-Table 1). The protocol was published in the PROSPERO (International Prospective Register of Systematic Reviews database; registration number CRD42015029910).

### Study Selection

We used the Population-Exposure-Outcome-Study Design criteria throughout the review process, based on type of participants, type of exposure, type of outcome, and study design.

#### Type of Participants

All studies of adults aged 18 years and over were eligible for inclusion in this review.

#### Type of Exposure

We included all studies that had assessed alcohol consumption, either by self-report or a proxy such as clinical records, defined either as drinking level (low, moderate, heavy, alcohol abuse, alcoholism) or as frequency (grams per day).

#### Type of Outcome

The outcome of interest was ARDS. We excluded studies limited to specific clinical diagnoses (HIV, hepatitis B and C viruses).

#### Study Design

All the primary comparative observational studies were included (longitudinal/cohort, case control, cross sectional).

### Search Strategy

Medline (via Ovid), EMBASE (via Ovid), and Web of Science were searched independently by two authors from December 1985 to December 2015. Search filters for observational study designs were used,^15^ and search terms for both outcome and exposure were developed from relevant Cochrane Reviews groups^16^ (e-Table 2). The search terms using every possible combination were the following: Respiratory Distress Syndrome, Adult/or Adult Respiratory Distress Syndrome/or Acute Lung Injury/or Acute Respiratory Distress Syndrome/or ARDS or ALI. The reference lists were also screened in order to identify additionally eligible studies. There was no language limitation, and where necessary translations of foreign language articles were conducted. In case of duplication the most informative study was used. Two reviewers (E. S., J. L.-B.) independently screened the titles and abstracts. All relevant studies were obtained and the full text was screened independently by two reviewers (E. S., J. L.-B.). Any disagreements were resolved through discussion or with the help of the third reviewer (J. B.).

### Data Extraction

The data extraction was performed independently by two reviewers, using a previous pilot data extraction form. Variables of interest included author, year of study, study design, definitions of exposure (alcohol) and outcome (ARDS), geographic location, reference population, demographic of study population setting, number of people recruited, and adjustment for confounders.

For categorical measures of alcohol drinking, where possible we compared any alcohol vs no alcohol consumption (reference group). When the nonalcohol category was not reported in the studies, the lowest exposed category was used as the reference group. Where exposure to alcohol was reported as quantiles or as categories, we compared the highest exposure groups with lowest exposed group. Also, in the analysis, categorical measures of alcohol consumption were further defined as levels of consumption: light/moderate/heavy drinking; alcohol abuse (including alcoholism). Grams of daily alcohol consumption were used as a standard measure, defining one drink as 0.6 ounce, 14.0 g, or 1.2 tablespoons of pure alcohol.^17^ According to the Centers for Disease Control and Prevention guidelines, we defined heavy drinking as a weekly consumption of 15 or more drinks for men, and eight or more drinks for women, whereas binge drinking was defined either as five or more drinks during a single occasion for men, and four or more for women. Excessive drinking was defined as the presence of either binge or heavy drinking.^17^ Moderate alcohol drinking was defined as the daily consumption of up to one drink for women and two drinks for men.^18^

### Assessment of Study Quality

The quality of the studies was assessed by the Newcastle-Ottawa Scale.^19^ High quality was defined as a grade of ≥ 6. Both case-control and cohort studies had a maximum score of 9; whereas cross-sectional studies had a score of 7. The quality assessment was not conducted for articles published as abstracts, due to the lack of information. Two reviewers (E. S., J. L.-B.) independently assessed the quality of the included studies. Discrepancies were resolved through discussion and consensus.

### Statistical Analysis

Relative measures of effect were estimated as odds ratios (ORs), relative risks (RRs), or hazard ratios (HRs) with 95% confidence intervals. Results were extracted as either adjusted effect measures, crude measures of effect, or using raw data. We used adjusted estimates in preference. Where more than one adjusted estimate was presented in the paper, we used the estimate that was adjusted for smoking and other socioeconomic factors, where available. For case-control studies we estimated the OR whereas for cohort and cross-sectional studies we estimated the RR. When alcohol exposure was reported either as quantiles or categories, we extracted the effect estimates, taking the highest vs the lowest exposure group. We pooled odds ratios and relative risks together in cases of a rare outcome. Also, studies assessing the effect of definite transfusion-related ALI were analyzed separately and thus not combined in the meta-analysis with other predisposing condition resulting in ALI.

Because of the anticipated heterogeneity between the studies, DerSimonian and Laird random-effects models were used to weight each study. The *I*^2^ statistic was used to indicate between the studies the percentage of variation due to heterogeneity.^20^ Subgroup analyses were carried out to explain the identified heterogeneity, based on predisposing condition for ARDS, study design, study quality, year of publication, geographic location, and adjustment for confounders. We used Egger’s statistical test for assessment of publication bias, and a funnel plot for visual assessment. Stata software version 14 (StataCorp) and Review manager software version 5.3 (Cochrane Collaboration) were both used for the statistical analysis. A *P* value < .05 was thought to represent a statistically significant level.

## Results

**Figure 1:**
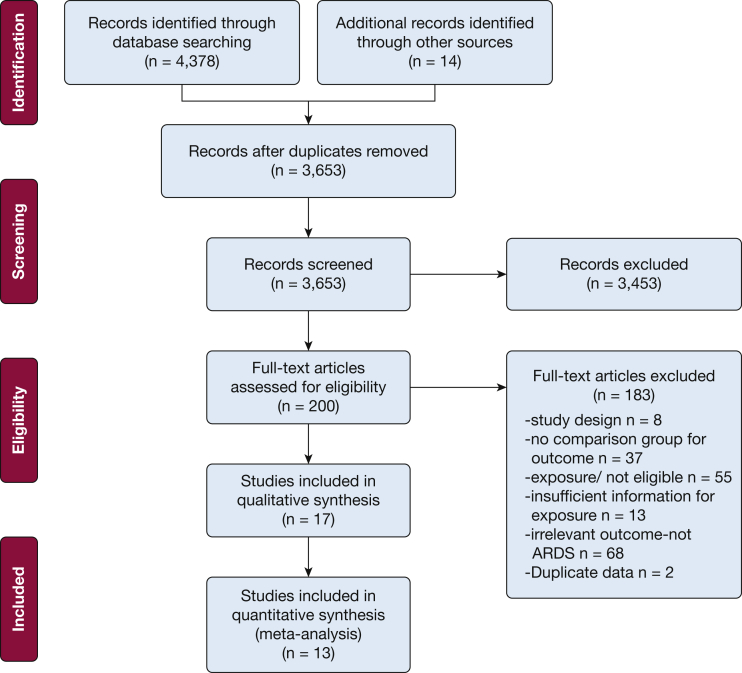
Flow chart of studies.

Database searches and reference lists yielded a total of 4,392 articles (Fig 1). After the removal of 739 duplicates we identified 3,653 articles for titles/abstracts screening, from which we identified 200 articles for full text review. Of these, 183 were excluded because the study design was a review or a letter (eight studies); or because there was no comparison group (37 studies); insufficient information on exposure and outcome (13 studies); ineligible outcomes such as sleep apnea, pneumonia, asthma, COPD, airway obstruction, oxygen desaturation index (68 studies); irrelevant exposure (55 studies); or duplicate data (two studies). Thus 17 studies met our criteria for inclusion in the review.

### Study Characteristics

The characteristics of the 17 included studies in the review are shown in Table 1. Twelve studies used a cohort design^21^, ^22^, ^23^, ^24^, ^25^, ^26^, ^27^, ^28^, ^29^, ^30^, ^31^, ^32^; four were case-control studies^33^, ^34^, ^35^, ^36^, and one was a cross-sectional study using survey data.^37^ A total population of 177,674 people was included. Patients with ARDS had a mean age ranging from 33 to 72.7 years, were more likely to be male (range, 50% to 85%; 13 studies), and the majority were white (range, 50% to 88%; eight studies).

**Table 1:** Characteristics of the Included Studies

Study/Year	Study Design	Country	Population/Main Predisposing Condition	Characteristics of Patients With ARDS	No. of People Included	Alcohol Ascertainment	Definition of Exposure to Alcohol	Definition Used to Ascertain ARDS	Adjustment
Afshar et al^21^/2014	Cohort	USA	Hospital/Trauma	Age: 33 y^a^ Male: 80.6% White: 57.7%	26,305	Blood alcohol content	> 0 mg/dL	Berlin	Adjusted for: age, sex, race, tobacco, diabetes mellitus, immunosuppression medication
Ahmed et al^33^/2014	Nested case control	USA	Hospital	Age: — Male: — White: —	828	…	Any use	…	Matched for: age, sepsis, sex, surgery, ratio of oxygen saturation to fraction of inspired oxygen, and lung injury prediction score
Calfee et al^22^/2011^b^	Cohort	USA	Hospital/Trauma	Age: 44 y Male: 81% White: 66%	144	AUDIT Questionnaire	Alcohol abuse	AECC	No adjustment/matching performed
Calfee et al^23^/2015	Cohort	USA	Hospital	Age: 56 y Male: 53% White: 88%	426	AUDIT Questionnaire	Alcohol abuse	AECC	Adjusted for: log-NNAL, APACHE II scores, race, diabetes, time elapsed between admission and enrollment
Cardinal-Fernandez et al^24^/2013	Cohort	Europe	Hospital/Sepsis	Age: 57 y Male: 71.4% White: —	149	Questionnaire	Alcoholism	AECC	No adjustment/matching performed
Gajic et al^34^/2007^b^	Nested case control	USA	Hospital/ICU	Age: 61 y^a^ Male: 50% White: —	74	Medical records	Alcohol abuse	AECC	Matched for: age, sex, and admission diagnosis
Gajic et al^25^/2011^b^	Cohort	USA	Hospital	Age: 57 y^a^ Male: 65% White: 60%	5,584	Questionnaire	Alcohol abuse	AECC	Adjusted for predisposing conditions, high-risk surgery, high-risk trauma, male sex, body mass index, chemotherapy, diabetes, smoking, emergency surgery, tachypnea, hypoalbuminemia, acidosis, Spo_2_, Fio_2_
Ge et al^26^/2014	Cohort	China	Hospital/ICU	Age: — Male: — White: —	343	Questionnaire	Alcohol abuse	AECC	Adjusted for: age, sex, smoking, use of alcohol, history of diabetes, sepsis, septic shock, trauma, pneumonia, aspiration, massive blood transfusion, bacteremia, pulmonary contusion
Iribarren et al^27^/2000	Cohort	USA	Hospital	Age: 52.8 y Male: 59% White: 73%	121,012	Questionnaire	≥ 3 drinks/d in previous year	AECC	Adjusted for: age, sex, race, smoking, body mass index, education
Iscimen et al^28^/2008^b^	Cohort	Europe	Hospital/Septic shock	Age: — Male: — White: —	160	Medical records	Alcohol abuse	…	Adjusted for: delayed goal-directed resuscitation, delayed antibiotics, chemotherapy, transfusion, diabetes mellitus
Kojicic et al^35^/2012^b^	Case control	USA	Hospital/Pneumonia	Age: 64.5 y^a^ Male: 50% White: —	596	Medical records	Alcohol abuse	AECC	Matched for: specific pathogen, isolation site, sex, and age
Licker et al^29^/2003^b^	Cohort	USA	Hospital	Age: 67 y Male: — White: —	869	Medical records	Alcohol abuse > 60 g/d	AECC	Adjusted for: pneumonectomy, ventilator hyperpressure index, fluid infused
Moss et al^30^/1996	Cohort	USA	Hospital/Sepsis, trauma	Age: 45.2 y Male: 63% White: 50%	351	Medical records	Alcohol abuse	AECC	Adjusted for: sex, at- risk diagnosis, APACHE II score
Moss et al^31^/2003	Cohort	USA	Hospital/Septic shock	Age: 50.1 y Male: 68% White: —	220	SMAST Questionnaire	Alcohol abuse	AECC	Adjusted for: source of infection, sex, age, chronic hepatic dysfunction, diabetes, severity of illness, nutritional status, and smoking status
TenHoor et al^37^/2001	Cross sectional	USA	Hospital/Decedents	Age: 72.7 y Male: 51% White: 86%	19,003	Interview	≥ 3 drinks/wk	Death certificate	Adjusted for: sepsis, cirrhosis, medical or surgical misadventure, injury, nonwhite, male, age > 64 y, current smoking/former smoking
Thakur et al^32^/2009	Cohort	USA	Hospital/ICU	Age: 55 y Male: 85% White: —	1,357	Interview	> 14 drinks/wk	AECC	Adjusted for: aspiration, chemotherapy, high-risk surgery, pancreatitis, sepsis, shock, smoking, cirrhosis, and sex
Toy et al^36^/2012^b^	Case control	USA	Hospital	Age: 54 y Male: 49% White: 71%	253	Medical records	Alcohol abuse	AECC	No adjustment/matching performed

AECC = American-European Consensus Conference definition; APACHE II = Acute Physiology and Chronic Health Evaluation II; AUDIT = Alcohol Use Disorders Identification Test; Fio_2_ = fraction of inspired oxygen; log-NNAL = log-transformed NNAL [4-(methylnitrosamino)-1-(3-pyridyl)-1-butanol] level; SMAST = Short Michigan Alcohol Screening Test; Spo_2_ = oxygen saturation as measured by pulse oximetry.

a Median presented.

b Outcome definition used within the study is acute lung injury.

All studies were conducted in a hospital setting, with 14 being conducted in the United States, two in Europe,^24^, ^28^ and one in China.^26^ Fourteen studies adjusted for confounders^21^, ^23^, ^25^, ^26^, ^27^, ^28^, ^29^, ^30^, ^31^, ^32^, ^33^, ^34^, ^35^, ^37^ and seven of these had reported results adjusted for smoking.

Study quality was assessed using the Newcastle-Ottawa Scale for 15 studies (two studies were published as an abstract only) and of these, eight (53.3%) were found to be of high quality. The median risk of bias score was 6, indicating a medium risk of bias (Table 2). The main reasons for lower scores in risk of bias were as follows: flawed study design (lack of objective/validated methods for exposure definition), selection bias (representativeness of sample population) and information bias (lack of provided information description in outcome assessment), or nonadequacy of follow-up.

**Table 2:** Critical Appraisal of the Included Studies, Using Newcastle-Ottawa Scale

Study/Year	No. of Stars			
	Selection^a^	Comparability^b^	Exposure^c^	Overall Score
Afshar et al^21^/2014	3	2	3	8
Ahmed et al^33^/2014^d^	…	…	…	…
Calfee et al^22^/2011	3	0	2	5
Calfee et al^23^/2015	3	1	2	6
Cardinal-Fernandez et al^24^/2013	1	0	3	4
Gajic et al^34^/2007	2	1	1	4
Gajic et al^25^/2011	2	0	2	4
Ge et al^26^/2014	2	2	3	7
Iribarren et al^27^/2000	2	2	2	6
Iscimen et al^28^/2008^d^	…	…	…	…
Kojicic et al^35^/2012	2	1	1	4
Licker et al^29^/2003	2	1	3	6
Moss et al^31^/2003	2	2	3	7
Moss et al^30^/1996	1	1	2	4
TenHoor et al^37^/2001	2	2	2	6
Thakur et al^32^/2009	2	2	2	6
Toy et al^36^/2012	2	0	1	3

a Maximum, four stars.

b Maximum, two stars.

c Maximum, three stars.

d Only abstract available—not quality assessment.

### Exposure Reporting

Sixteen studies investigated the effects of chronic alcohol exposure, and one the effect of acute exposure assessed by blood alcohol levels.^21^ Most of the studies reported chronic alcohol exposure assessed alcohol by self-report from a questionnaire^22^, ^23^, ^24^, ^25^, ^26^, ^27^, ^31^ or interview^32^, ^37^; six used alcohol consumption documented in medical records^28^, ^29^, ^30^, ^34^, ^35^, ^36^ and in one study the method of assessment and the definition of alcohol consumption were not defined.^33^ Measures of alcohol consumption included drinks per day,^27^ drinks per week,^32^, ^37^ milligrams of alcohol per deciliter of blood,^21^ alcoholism,^24^ and alcohol abuse ascertained either from medical records or questionnaire.^22^, ^23^, ^25^, ^26^, ^28^, ^29^, ^30^, ^31^, ^34^, ^35^, ^36^ Specifically, alcohol abuse was defined in three of the 11 studies using a validated questionnaire, two defined alcohol abuse using the AUDIT (Alcohol Use Disorders Identification Test),^22^, ^23^ and one using the SMAST (Short Michigan Alcohol Screening Test).^31^ All studies analyzed the effects of alcohol exposure as a binary measure, contrasting high with low intake, or a history of abuse with no history of abuse, or any alcohol intake with none.

### Outcome Reporting

Outcome definitions for ARDS included the American-European Consensus Conference definition,^22^, ^23^, ^24^, ^25^, ^26^, ^27^, ^29^, ^30^, ^31^, ^32^, ^34^, ^35^, ^36^ death certificates,^37^ and the Berlin definition.^21^Two studies did not provide clear information on outcome definition.^28^, ^33^

### Meta-Analysis

**Figure 2:**
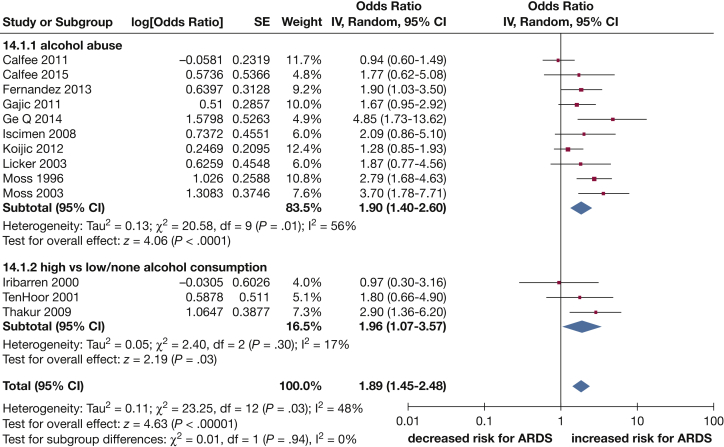
Forest plot of alcohol consumption and the risk of ARDS; subgroup analysis based on alcohol abuse vs high alcohol consumption.

**Figure 3:**
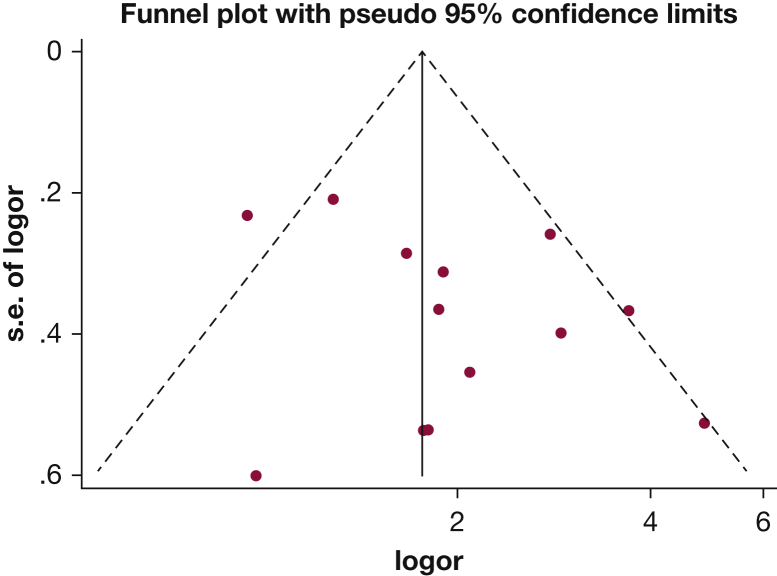
Funnel plot of any high alcohol consumption and the risk of ARDS.

Thirteen of the studies provided data that could be included in a pooled analysis, which demonstrated that any measure of high exposure to alcohol significantly increased the risk of ARDS by a ratio of 1.89 (95% CI, 1.45-2.48; *I*^2^ = 48%) (Fig 2). No evidence of publication bias was found (funnel plot [Fig 3 and Egger’s asymmetry test], *P* = .150).

Similar magnitudes of increased risk were seen in sensitivity analyses limited to studies categorizing alcohol intake as alcohol abuse (OR, 1.90; 95% CI, 1.40-2.60; *I*^2^ = 56%) (Fig 2), and limited to studies comparing only high alcohol with low or no alcohol consumption (OR, 1.96; 95% CI, 1.07-3.57; *I*^2^ = 17%) (Fig 2). However, the only study to use a zero intake as the reference group^27^ found no significant effect of consuming of ≥ 3 drinks per day during the last year (OR, 0.97; 95% CI, 0.30-3.16). A further sensitivity analysis excluding one study, which compared decedents with a diagnosis of ARDS compared with decedents with other diagnoses,^37^ had a marginal effect on the magnitude of the association (OR, 1.91; 95% CI, 1.43-2.54; 12 studies) compared with the unrestricted analysis.

**Figure 4:**
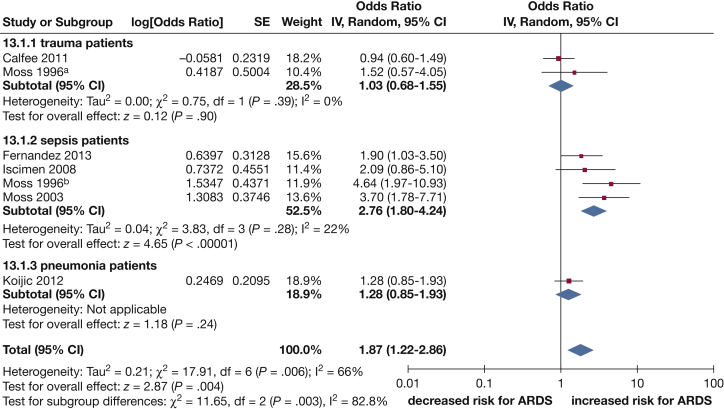
Forest plot of alcohol consumption and the risk of ARDS; subgroup analysis in patients with trauma, sepsis, and pneumonia. ^a^Data presented for the subset of trauma patients; ^b^Data presented for the subset of sepsis patients.

Subgroup analysis found that the predisposing condition (trauma, sepsis/septic shock, pneumonia) for ARDS explained heterogeneity between the studies (*P* value for subgroup differences, .003); where an increased risk of ARDS associated with alcohol consumption was apparent only in patients with sepsis/septic shock (OR, 2.76; 95% CI, 1.80-4.24; four studies) (Fig 4). Further analyses to explore reasons for heterogeneity in the meta-analysis (e-Table 3) showed no statistically significant interaction by study design (case control, longitudinal/cohort, cross sectional; *P* = .22), study quality (high vs low; *P* = .09), country of study (United States, Europe, China; *P* = .19), effect estimate (adjusted vs unadjusted analysis; *P* = .21), and year of publication (1995-2005 vs 2006-2015; *P* = .20).

Two studies were identified that assessed the effects of alcohol on the risk of transfusion-related ALI.^34^, ^36^ Both studies found that alcohol increased the risk of transfusion-related ALI (results: *P* = .006 [37% vs 18%]; OR, 3.0; 95% CI, 1.07-8.7). A meta-analysis of these two studies could not be performed as the first study^34^ did not provide sufficient information to allow ORs to be estimated, due to the study using individual matching to identify the control subjects. Two further studies could not be included in the meta-analysis. The first of these compared risks of ARDS in those with alcohol detected in blood compared with those with no detectable alcohol^21^; as the effects of acute alcohol intoxication are very different from those of chronic alcohol exposure, this study was not included in the meta-analysis. This study found that the presence of alcohol in blood was associated with an increased risk of ARDS (OR, 1.50). The second study was published only in abstract form,^33^ which did not provide sufficient information to allow ORs to be estimated, due to the study using individual matching. Briefly, this study showed that patients with ARDS were more likely to consume alcohol (17% vs 10%) compared with control subjects.

## Discussion

This article reports the first meta-analysis of observational studies of the association between alcohol consumption and the risk of ARDS among adults. We found evidence of a 1.89-fold increase in the odds of ARDS in persons with high alcohol consumption, which in subgroup analyses appeared to be attributable to the effect of exposure defined as alcohol abuse and also in those with sepsis or septic shock as the predisposing condition for ARDS.

Our review is based on a comprehensive search of the worldwide literature held in key medical databases and using search terms from recognized sources, complemented by searches of reference lists from identified publications. We imposed no language restriction in our searches. It is therefore likely that our results are representative and generalizable. The absence of publication bias further validates our findings.

Being based largely on observational studies raises the possibility of bias, which may be introduced in our analysis. However, misclassification bias due to the inclusion of former/lower drinkers in the reference group is likely, if anything, to have reduced the magnitudes of estimated effects. However, the subgroup analyses were conducted in an attempt to explore reasons for heterogeneity, and we found that there were no significant differences according to study quality, study design, effect estimate, continent, or year of publication.

A previous narrative review has drawn attention to the potential importance of chronic alcohol abuse in the etiology of ARDS,^38^ finding an increased incidence of ARDS in alcohol abusers. Also, a narrative review published in 2009, which included only four studies on alcohol and ARDS, concluded that alcohol abuse is a risk factor for the development of ARDS.^7^ Our findings extend the conclusions of this work, identifying a summary effect estimate and that the increased risk applies predominantly to ARDS arising from sepsis.

The mechanism or mechanisms by which alcohol consumption might increase the risk of ARDS, particularly among patients with sepsis, are not fully understood. However, effects on membrane permeability,^39^, ^40^ glutathione depletion,^41^, ^42^, ^43^ Toll-like receptor up-regulation,^44^ expression of transforming growth factor-β_1_,^45^, ^46^ and impairment of macrophage function are all potential explanations.^47^

Our study thus provides comprehensive evidence that high alcohol consumption increases the risk of ARDS.

## Supplementary Data

e-Online Data

## References

[1] Thompson B.T., Chambers R.C., Liu K.D. (2017). Acute respiratory distress syndrome. N Engl J Med.

[2] Bellani G., Laffey J.G., Pham T. (2016). Epidemiology, patterns of care, and mortality for patients with acute respiratory distress syndrome in intensive care units in 50 countries. JAMA.

[3] Henderson W.R., Chen L., Amato M.B., Brochard L.J. (2017). Fifty years of research in ARDS: respiratory mechanics in acute respiratory distress syndrome. Am J Respir Crit Care Med.

[4] Ware L.B., Matthay M.A. (2000). The acute respiratory distress syndrome. N Engl J Med.

[5] Phua J., Badia J.R., Adhikari N.K. (2009). Has mortality from acute respiratory distress syndrome decreased over time? A systematic review. Am J Respir Crit Care Med.

[6] Ranieri V., Rubenfeld G., Thompson B. (2012). Acute respiratory distress syndrome: the Berlin definition. JAMA.

[7] Boé D.M., Vandivier R.W., Burnham E.L., Moss M. (2009). Alcohol abuse and pulmonary disease. J Leukoc Biol.

[8] Umbrello M., Formenti P., Bolgiaghi L., Chiumello D. (2016). Current concepts of ARDS: a narrative review. Int J Mol Sci.

[9] Jia X., Malhotra A., Saeed M., Mark R.G., Talmor D. (2008). Risk factors for acute respiratory distress syndrome in patients mechanically ventilated for greater than 48 hours. Chest.

[10] Hudson L.D., Milberg J.A., Anardi D., Maunder R.J. (1995). Clinical risks for development of the acute respiratory distress syndrome. Am J Respir Crit Care Med.

[11] Laycock H., Rajah A. (2010). Acute lung injury and acute respiratory distress syndrome: a review article. Br J Med Practitioner.

[12] Mehta A. (2013). Pulmonary consequences of alcoholism: a critical review. OA Alcohol.

[13] Liberati A., Altman D.G., Tetzlaff J. (2009). The PRISMA statement for reporting systematic reviews and meta-analyses of studies that evaluate health care interventions: explanation and elaboration. PLoS Med.

[14] Stroup D.F., Berlin J.A., Morton S.C. (2000). Meta-analysis of observational studies in epidemiology: a proposal for reporting. JAMA.

[15] Scottish Intercollegiate Guidelines Network (SIGN). Search filters, observational studies. http://www.sign.ac.uk/search-filters.html. Accessed December 4, 2015.

[16] Cochrane Library. http://www.cochranelibrary.com/. Accessed December 4, 2015.

[17] Centers for Disease Control and Prevention (CDC). *Alcohol and Public Health: Fact Sheets: Alcohol Use and Your Health*. http://www.cdc.gov/alcohol/fact-sheets/alcohol-use.htm. Accessed December 10, 2015.

[18] U.S. Department of Health and Human Services and U.S. Department of Agriculture. 2015–2020. Dietary Guidelines for Americans. 8th Ed. Washington, DC; 2015. https://health.gov/dietaryguidelines/2015/resources/2015-2020_Dietary_Guidelines.pdf. Accessed December 15, 2015.

[19] Wells G, Shea B, O’Connell D, et al. The Newcastle-Ottawa Scale (NOS) for assessing the quality of nonrandomised studies in meta-analyses. http://www.ohri.ca/programs/clinical_epidemiology/oxford.asp. Accessed February 10, 2016.

[20] Higgins J., Thompson S.G. (2002). Quantifying heterogeneity in a meta-analysis. Stat Med.

[21] Afshar M., Smith G.S., Terrin M.L. (2014). Blood alcohol content, injury severity, and adult respiratory distress syndrome. J Trauma Acute Care Surg.

[22] Calfee C.S., Matthay M.A., Eisner M.D. (2011). Active and passive cigarette smoking and acute lung injury after severe blunt trauma. Am J Respir Crit Care Med.

[23] Calfee C.S., Matthay M.A., Kangelaris K.N. (2015). Cigarette smoke exposure and the acute respiratory distress syndrome. Crit Care Med.

[24] Cardinal-Fernandez P., Ferruelo A., El-Assar M. (2013). Genetic predisposition to acute respiratory distress syndrome in patients with severe sepsis. Shock.

[25] Gajic O., Dabbagh O., Park P.K., U.S. Critical Illness and Injury Trials Group: Lung Injury Prevention Study Investigators (USCIITG-LIPS) (2011). Early identification of patients at risk of acute lung injury: evaluation of lung injury prediction score in a multicenter cohort study. Am J Respir Crit Care Med.

[26] Ge Q., Yao Z., Wang T. (2014). [Risk factors of the occurrence and death of acute respiratory distress syndrome: a prospective multicenter cohort study] [article in Chinese]. Zhonghua Wei Zhong Bing Ji Jiu Yi Xue.

[27] Iribarren C., Jacobs D.R., Sidney S., Gross M.D., Eisner M.D. (2000). Cigarette smoking, alcohol consumption, and risk of ARDS: a 15-year cohort study in a managed care setting. Chest.

[28] Iscimen R., Cartin-Ceba R., Yilmaz M. (2008). Risk factors for the development of acute lung injury in patients with septic shock: an observational cohort study. Crit Care Med.

[29] Licker M., de Perrot M., Spiliopoulos A. (2003). Risk factors for acute lung injury after thoracic surgery for lung cancer. Anesth Analg.

[30] Moss M., Bucher B., Moore F.A., Moore E.E., Parsons P.E. (1996). The role of chronic alcohol abuse in the development of acute respiratory distress syndrome in adults. JAMA.

[31] Moss M., Parsons P.E., Steinberg K.P. (2003). Chronic alcohol abuse is associated with an increased incidence of acute respiratory distress syndrome and severity of multiple organ dysfunction in patients with septic shock. Crit Care Med.

[32] Thakur L., Kojicic M., Thakur S.J. (2009). Alcohol consumption and development of acute respiratory distress syndrome: a population-based study. Int J Environ Res Public Health.

[33] Ahmed A., Biehl M., Kashyap R., Hanson A.C., Schenck L.A., Gajic O. (2014). The impact of acute respiratory distress syndrome (ARDS) on short and long-term survival: a population-based nested case-control study. Am J Respir Crit Care Med.

[34] Gajic O., Rana R., Winters J.L. (2007). Transfusion-related acute lung injury in the critically ill: prospective nested case-control study. Am J Respir Crit Care Med.

[35] Kojicic M., Li G.X., Hanson A.C. (2012). Risk factors for the development of acute lung injury in patients with infectious pneumonia. Crit Care.

[36] Toy P., Gajic O., Bacchetti P. (2012). Transfusion-related acute lung injury: incidence and risk factors. Blood.

[37] TenHoor T., Mannino D.M., Moss M. (2001). Risk factors for ARDS in the United States: analysis of the 1993 National Mortality Followback Study. Chest.

[38] Moss M., Burnham E.L. (2003). Chronic alcohol abuse, acute respiratory distress syndrome, and multiple organ dysfunction. Crit Care Med.

[39] Burnham E.L., Halkar R., Burks M., Moss M. (2008). The effects of alcohol abuse on pulmonary alveolar-capillary barrier function in humans. Alcohol Alcohol.

[40] Fan X., Joshi P.C., Koval M., Guidot D.M. (2011). Chronic alcohol ingestion exacerbates lung epithelial barrier dysfunction in HIV-1 transgenic rats. Alcohol Clin Exp Res.

[41] Guidot D.M., Modelska K., Lois M. (2000). Ethanol ingestion via glutathione depletion impairs alveolar epithelial barrier function in rats. Am J Physiol Lung Cell Mol Physiol.

[42] Velasquez A., Bechara R.I., Lewis J.F. (2002). Glutathione replacement preserves the functional surfactant phospholipid pool size and decreases sepsis-mediated lung dysfunction in ethanol-fed rats. Alcohol Clin Exp Res.

[43] Yeh M.Y., Burnham E.L., Moss M., Brown L.A.S. (2008). Non-invasive evaluation of pulmonary glutathione in the exhaled breath condensate of otherwise healthy alcoholics. Respir Med.

[44] Bailey K.L., Romberger D.J., Katafiasz D.M. (2015). TLR2 and TLR4 expression and inflammatory cytokines are altered in the airway epithelium of those with alcohol use disorders. Alcohol Clin Exp Res.

[45] Curry-McCoy T.V., Venado A., Guidot D.M., Joshi P.C. (2013). Alcohol ingestion disrupts alveolar epithelial barrier function by activation of macrophage-derived transforming growth factor beta1. Respir Res.

[46] Sueblinvong V., Kerchberger V.E., Saghafi R., Mills S.T., Fan X., Guidot D.M. (2014). Chronic alcohol ingestion primes the lung for bleomycin-induced fibrosis in mice. Alcohol Clin Exp Res.

[47] Joshi P.C., Mehta A., Jabber W.S., Fan X., Guidot D.M. (2009). Zinc deficiency mediates alcohol-induced alveolar epithelial and macrophage dysfunction in rats. Am J Respir Cell Mol Biol.

