# Supplementary material for: The Effect of Alcohol Consumption on the Risk of ARDS: A Systematic Review and Meta-Analysis
Source: Chest. 2017 Dec 27;154(1):58–68. doi: 10.1016/j.chest.2017.11.041 (PMC6045784; doi:10.1016/j.chest.2017.11.041)
Supplement: e-Online Data [file mmc1.pdf]

CHEST Online Supplement

The Effect of Alcohol Consumption on the Risk of ARDS

A Systematic Review and Meta-Analysis

Evangelia Simou, MSc; Jo Leonardi-Bee, PhD; and John Britton, MD

CHEST 2018; 154(1):58-68

Online supplements are not copyedited prior to posting and the author(s) take full responsibility for the accuracy of all data.

© 2018 AMERICAN COLLEGE OF CHEST PHYSICIANS. Reproduction of this article is prohibited without written permission from the American College of Chest Physicians. See online for more details. **DOI:** 10.1016/j.chest.2017.11.041

CHEST Online Supplement

e-Table 1: PRISMA checklist

Section/topic	#	Checklist item	Reported on page #
**TITLE**			
Title	1	Identify the report as a systematic review, meta-analysis, or both.	1
**ABSTRACT**			
Structured summary	2	Provide a structured summary including, as applicable: background; objectives; data sources; study eligibility criteria, participants, and interventions; study appraisal and synthesis methods; results; limitations; conclusions and implications of key findings; systematic review registration number.	1
**INTRODUCTION**			
Rationale	3	Describe the rationale for the review in the context of what is already known.	2
Objectives	4	Provide an explicit statement of questions being addressed with reference to participants, interventions, comparisons, outcomes, and study design (PICOS).	2
**METHODS**			
Protocol and registration	5	Indicate if a review protocol exists, if and where it can be accessed (e.g., Web address), and, if available, provide registration information including registration number.	3
Eligibility criteria	6	Specify study characteristics (e.g., PICOS, length of follow-up) and report characteristics (e.g., years considered, language, publication status) used as criteria for eligibility, giving rationale.	3
Information sources	7	Describe all information sources (e.g., databases with dates of coverage, contact with study authors to identify additional studies) in the search and date last searched.	4
Search	8	Present full electronic search strategy for at least one database, including any limits used, such that it could be repeated.	4
Study selection	9	State the process for selecting studies (i.e., screening, eligibility, included in systematic review, and, if applicable, included in the meta-analysis).	4
Data collection process	10	Describe method of data extraction from reports (e.g., piloted forms, independently, in duplicate) and any processes for obtaining and confirming data from investigators.	4
Data items	11	List and define all variables for which data were sought (e.g., PICOS, funding sources) and any assumptions and simplifications made.	4
Risk of bias in individual studies	12	Describe methods used for assessing risk of bias of individual studies (including specification of whether this was done at the study or outcome level), and how this information is to be used in any data synthesis.	5
Summary measures	13	State the principal summary measures (e.g., risk ratio, difference in means).	6
Synthesis of results	14	Describe the methods of handling data and combining results of studies, if done, including measures of consistency (e.g., I^2) for each meta-analysis.	6

Online supplements are not copyedited prior to posting and the author(s) take full responsibility for the accuracy of all data.

Section/topic	#	Checklist item	Reported on page #
Risk of bias across studies	15	Specify any assessment of risk of bias that may affect the cumulative evidence (e.g., publication bias, selective reporting within studies).	6
Additional analyses	16	Describe methods of additional analyses (e.g., sensitivity or subgroup analyses, meta-regression), if done, indicating which were pre-specified.	6
**RESULTS**			
Study selection	17	Give numbers of studies screened, assessed for eligibility, and included in the review, with reasons for exclusions at each stage, ideally with a flow diagram.	7
Study characteristics	18	For each study, present characteristics for which data were extracted (e.g., study size, PICOS, follow-up period) and provide the citations.	8
Risk of bias within studies	19	Present data on risk of bias of each study and, if available, any outcome level assessment (see item 12).	9
Results of individual studies	20	For all outcomes considered (benefits or harms), present, for each study: (a) simple summary data for each intervention group (b) effect estimates and confidence intervals, ideally with a forest plot.	13
Synthesis of results	21	Present results of each meta-analysis done, including confidence intervals and measures of consistency.	12
Risk of bias across studies	22	Present results of any assessment of risk of bias across studies (see Item 15).	12
Additional analysis	23	Give results of additional analyses, if done (e.g., sensitivity or subgroup analyses, meta-regression [see Item 16]).	12
**DISCUSSION**			
Summary of evidence	24	Summarize the main findings including the strength of evidence for each main outcome; consider their relevance to key groups (e.g., healthcare providers, users, and policy makers).	14
Limitations	25	Discuss limitations at study and outcome level (e.g., risk of bias), and at review-level (e.g., incomplete retrieval of identified research, reporting bias).	14
Conclusions	26	Provide a general interpretation of the results in the context of other evidence, and implications for future research.	15
**FUNDING**			
Funding	27	Describe sources of funding for the systematic review and other support (e.g., supply of data); role of funders for the systematic review.	1

Online supplements are not copyedited prior to posting and the author(s) take full responsibility for the accuracy of all data.

e-Table 2: Medline (via Ovid), EMBASE (via Ovid) and Web of Science search terms for primary studies

**Medline via Ovid search terms**
1. Epidemiologic studies/
2. Exp case control studies/
3. Exp cohort studies/
4. Case control.tw.
5. (cohort adj (study or studies)).tw.
6. Cohort analy$.tw.
7. (Follow up adj (study or studies)).tw.
8. (observational adj (study or studies)).tw.
9. Longitudinal.tw.
10. Retrospective.tw.
11. Cross sectional.tw.
12. Cross-sectional studies/
13. Or/1-12
14. exp Alcohol-Related Disorders/
15. Alcohol Drinking/
16. (alcohol adj3 (drink$ or intoxicat$ or use$ or abus$ or misus$ or risk$ or consum$ or withdraw$ or detox$ or treat$ or therap$ or excess$ or reduc$ or cessation or intervention$)).tw.
17. (drink$ adj3 (excess or heavy or heavily or harm or harmful or hazard$ or binge or problem$)).tw.
18. alcoholic$.tw.
19. 14 or 15 or 16 or 17 or 18
20. Respiratory Distress Syndrome, Adult/ or Adult Respiratory Distress Syndrome/ or Acute Lung Injury/ or Acute Respiratory Distress Syndrome/ or ARDS.mp. or ALI.mp.
21. exp tidal volume/ or exp respiration, artificial/ or tidal volume.mp. or (protective adj3 ventilat*).mp. or (pressure* adj3 limited*).mp. or LPVS.mp.
22. 20 or 21
23. 13 and 19 and 22
**Embase via Ovid search terms**
1. Clinical study/
2. Case control study
3. Family study/
4. Longitudinal study/

Online supplements are not copyedited prior to posting and the author(s) take full responsibility for the accuracy of all data.

5. Retrospective study/
6. Prospective study/
7. Randomized controlled trials/
8. 6 not 7
9. Cohort analysis/
10. (Cohort adj (study or studies)).mp.
11. (Case control adj (study or studies)).tw.
12. (follow up adj (study or studies)).tw.
13. (observational adj (study or studies)).tw.
14. (epidemiologic$ adj (study or studies)).tw.
15. (cross sectional adj (study or studies)).tw.
16. Or/1-5,8-15
17. substance-related disorders/
18. ((drug or substance) adj (Addict$ or abus$ or dependen$)).mp
19. (intoxicat$ or abstinen$ or withdrawal$).mp.
20. (excessive$ adj use$).mp.
21. (use$ adj disorder$).mp.
22. (drinking adj behavi$3).mp.
23. drinking behavior.mp.
24. alcohol$.mp.
25. alcoholism/
26. (alcohol adj abuse).mp
27. 17 or 18 or 19 or 20 or 21 or 22 or 23 or 24 or 25 or 26
28. respiratory distress syndrome, adult/
29. ((acute adj lung adj injur:) or (shock adj lung) or ards).tw.
30. ((acute or adult) and (respiratory adj distress)).tw.
31. or/28-30
32. 16 and 27 and 31
**Web of Science search terms**
(alcohol* OR alcoholic beverage OR alcohol consumption OR alcohol drinking OR alcohol use OR alcohol intake OR alcoholism OR alcohol abuse OR ethanol* OR ethanol concentration) AND (respiratory distress syndrome OR Acute respiratory distress syndrome OR Adult respiratory distress syndrome) AND (longitudinal * OR case control* OR Cohort* OR case-control OR observational).

e-Table 3: Exploration of heterogeneity for association between alcohol consumption and risk of CAP

Factor	Pooled RR (95% CI)	I^2	P value for subgroup differences
**Overall result**	1.89 [1.45, 2.48]	48%	-
**Study design**			0.22
Case control	1.28 [0.85, 1.93]	-	
Cohort	2.02 [1.48, 2.77]	51%	
Cross sectional	1.80 [0.66, 4.90]	-	
**Methodological quality**			0.09
High quality (>6)	2.46 [1.71, 3.53]	8%	
Low quality (<6)	1.57 [1.08, 2.28]	64%	
**Geographic location**			0.19
USA	1.78 [1.30, 2.43]	53%	
Asia	4.85 [1.73, 13.62]	-	
Europe	1.96 [1.18, 3.24]	0%	
**Effect estimate**			0.21
Adjusted analysis	2.08 [1.59, 2.72]	32%	
Unadjusted analysis	1.30 [0.66, 2.56]	69%	
**Publication year**			0.20
Publication year: 1995-2005	2.44 [1.54, 3.86]	28%	
Publication year: 2006-2015	1.71 [1.27, 2.30]	42%	
**Predisposing condition**			0.003
Trauma	1.03 [0.68, 1.55]	0%	
Sepsis patients	2.76 [1.80, 4.24]	22%	
Pneumonia patients	1.28 [0.85, 1.93]	-	

Online supplements are not copyedited prior to posting and the author(s) take full responsibility for the accuracy of all data.
